# A Multi-Task Causal Knowledge Fault Diagnosis Method for PMSM-ITSF Based on Meta-Learning

**DOI:** 10.3390/s25041271

**Published:** 2025-02-19

**Authors:** Ping Lan, Liguo Yao, Yao Lu, Taihua Zhang

**Affiliations:** 1 School of Mechanical and Electrical Engineering, Guizhou Normal University, Guiyang 550025, China; 18302609547@163.com (P.L.); lgyao@gznu.edu.cn (L.Y.);; 2 Technical Engineering Center of Manufacturing Service and Knowledge Engineering, Guizhou Normal University, Guiyang 550025, China

**Keywords:** fault diagnosis, industrial robots, meta-learning, multi-task learning, causal knowledge

## Abstract

In the process of diagnosing the inter-turn short circuit fault of the joint permanent magnet synchronous motor of an industrial robot, due to the small and sparse fault sample data, it is easy to misdiagnose, and it is difficult to quickly and accurately evaluate the fault degree, lock the fault location, and track the fault causes. A multi-task causal knowledge fault diagnosis method for inter-turn short circuits of permanent magnet synchronous motors based on meta-learning is proposed. Firstly, the variation of parameters under the motor’s inter-turn short circuit fault is thoroughly investigated, and the fault characteristic quantity is selected. Comprehensive simulations are conducted using Simulink, Simplorer, and Maxwell to generate data under different inter-turn short circuit fault states; meanwhile, the sample data are accurately labeled. Secondly, the sample data are introduced into the learning network for training, and the multi-task synchronous diagnosis of the fault degree and position of the short circuit between turns is realized. Finally, the Neo4j database based on causality knowledge of motor inter-turn short circuit fault is constructed. Experiments show that this method can diagnose the fault location, fault degree, and fault cause of the motor with different voltage unbalanced degrees. The diagnosis accuracy of fault degree is 99.75 ± 0.25%, and the diagnosis accuracy of fault location and fault degree is 99.45 ± 0.21%.

## 1. Introduction

The Permanent Magnet Synchronous Machine (PMSM), with its outstanding performance—including high efficiency, high torque, high power factor, and precise motion control and positioning—enables industrial robots to maintain a high degree of flexibility and accuracy when performing complex tasks. It has become the mainstream in the field of end-effector applications [1]. Inter-turn short fault (ITSF) is a common short circuit problem in PMSM stator windings; due to the aging of insulation materials, external physical damage, or defects in manufacturing, the phenomenon of unexpected electrical connection between adjacent turns inside the winding [2] will induce circulation inside the coil and cause overheating, which will further accelerate the deterioration of the insulation layer of the surrounding wire and make the number of short circuit turns continue to rise [3].

The stable operation of the motor is seriously threatened [4], and it is difficult to ensure the normal operation of industrial robots.

There are three modern motor fault diagnosis methods: diagnosis method based on a mathematical model, diagnosis method based on signal processing, and diagnosis method based on artificial intelligence. The fault diagnosis method establishes the mathematical model of the specific motor fault according to the dynamic change of the physical attribute parameters of the motor [5]. The commonly used methods include the parameter identification method [6], the Kalman filter algorithm [7], and so on. References [8,9] identify the occurrence of faults using impedance changes caused by motor ITSF. In reference [10], the extended Kalman filter algorithm is used to collect the observed and estimated values of the PMSM fault model and predict the actual value to recognize the motor’s running state. Reference [11] proposes a BEMF estimation method for real-time identification of ITSF faults by back electromotive force under non-stationary and nonlinear states [12]. This method has a high precision for motor model establishment. It is sensitive to system faults while relatively insensitive to irrelevant variables of system input, ensuring faults in different states can be accurately identified [13]. However, constructing the mathematical model is more complicated, the training time is long, and the calculation amount is large, so it is not used in the fault diagnosis scenario with high real-time requirements.

The signal processing-based fault diagnosis method no longer relies on the complex input–output model of dynamic datasets. Instead, it revolves around the correlation between faults and signal patterns, based on the principle that faults directly impact output. To realize PMSM-ITSF fault diagnosis, attribute variable information is comprehensively analyzed across time, frequency, and time–frequency domains. Common signal processing methods include wavelet transform [14], short-time Fourier transform (STFT) [15], and motor current spectrum analysis [16]. At present, frequency domain and time–frequency domain analysis methods are widely used in the diagnosis of PMSM and fault diagnosis by observing and analyzing the third harmonic component [17] of the three-phase current of the motor stator. Principal component analysis [18], root mean square or mean value [19], kurtosis [20], and so on have become the mainstream methods. In reference [21], according to the weak correlation between the excitation current and the square of the vibration signal, the characteristics are significantly enhanced in the motor’s early fault stage, and the residual difference between the actual value and the predicted value of the excitation current is integrated to realize the fault diagnosis of PMSM-ITSF. Reference [22] uses Park transform analyses of fault components to determine fault characteristics. The transformed signal is used as a fault indicator to detect short circuit faults in PMSM stator windings [23], and the fault type is determined by its amplitude. In addition, a stator detection coil system is the proposed reference [24], which can find the relationship between detection signal amplitude value and fault severity when the fault dataset is small [25]. At the same time, there are many excellent PMSM-ITSF fault diagnosis methods, such as the instantaneous power decomposition method [26] and the harmful sequence impedance method [27]. Compared with single-feature fault diagnosis, the above method has higher diagnostic accuracy and can eliminate the influence of dynamic processes on motor fault characteristics. However, this algorithm’s operation needs to be based on better prior knowledge of motor faults. When the process is too complex to be accurately modeled by analysis, traditional signal analysis methods cannot diagnose precisely. Asset administration shells (AAS) are the core concept in Industry 4.0, used to describe and manage the digital models of physical assets [28]. AAS integrate the full life cycle data of assets to support various applications such as fault diagnosis, predictive maintenance, and asset management. In multi-agent systems (MAS), agents can share data with various devices or subsystems and collaborate with each other. They can monitor the operating status of specific devices in real time and update the data dynamically to the AAS [29]. When a certain device malfunctions, the relevant intelligent agents can quickly identify the problem and initiate the diagnostic process. Through the collaboration among intelligent agents, they can analyze complex faults and optimize the usage and maintenance plans of assets. In MAS, intelligent agents can integrate multi-source data (such as sensor data, historical fault records, etc.) and utilize machine learning algorithms to predict the remaining useful life (RUL) and potential faults of the equipment. Based on the prediction results, the intelligent agent can dynamically adjust the maintenance strategies, such as scheduling maintenance tasks in advance or replacing vulnerable components, thereby effectively avoiding sudden failures and enhancing the reliability and production efficiency of the equipment. Through the deep integration of MAS and AAS, the entire process, from data acquisition to fault diagnosis to maintenance decision-making, has been realized as fully intelligent.

In recent years, artificial intelligence has been widely used in fault diagnosis research due to its excellent performance in nonlinear fitting and strong anti-noise ability [30]. Because DL can dig out the outstanding performance of hidden deep information through sample data, applying DL to motor fault prediction and diagnosis is possible. Currently, the diagnosis of PMSM-ITSF mainly uses the combined feature quantity as the diagnostic basis for ITSF, which can effectively improve the diagnosis accuracy of motor faults [31]. Reference [32] proposed a PMSM-ITSF diagnosis method based on the combination of a cycle generative admissible network (Cycle GAN) [33], depth autoencoder, and depth autocoding (DAE) [34], which utilized the SOFTMAX classifier for accurate fault diagnosis with an accuracy of 98.73%.

During its whole life cycle, a PMSM is usually in a normal state because the motor’s ITSF diagnosis technology involves many fields of knowledge, such as mechanics and electromagnetism, and has the characteristics of strong concealability and rapid failure. Therefore, ITSF fault samples can be collected in small quantities and poorly. The simulation experiments effectively capture the dynamic characteristics of the motor under varying degrees of ITSF faults. These experiments not only circumvent the high costs and safety hazards associated with replicating diverse fault states and operating conditions in physical tests but also yield a substantial amount of experimental data within a limited timeframe, thereby providing robust data support for method validation. The simulation tool used has been widely used in the real-time monitoring of CNC machine tool operation based on digital twin [35], the residual service life prediction of rolling bearings based on digital twin and other related fields [36], and has been verified many times, and can reflect the characteristics of the actual system.

Because deep learning (DL) networks are prone to overfitting and false events in small sample datasets, obtaining a good PMSM-ITSF fault diagnosis accuracy is difficult. The original Gans take random noise as input very slowly, and the generated samples are of poor quality and are prone to mode breakdown [37]. Unlike traditional DL methods, meta-learning (ML) has a flexible framework, which can learn target tasks in limited datasets and use previous experience learning target tasks to quickly adapt to new tasks without starting from scratch [38]. As ML can learn under small sample datasets to obtain better diagnostic accuracy and has better generalization performance under new environments, it is becoming one of the most popular methods to solve zero-sum and small-sample learning problems [39]. Reference [40] proposes a diagnostic method based on multi-task learning meta-transfer learning, which aims to effectively learn knowledge from diverse tasks in parallel under limited data conditions, thus enhancing the overall learning efficiency. The paper [41] constructs a decentralized, federated meta-learning framework (DFMLF) for multi-tasking learning. The framework of DFMLF skillfully solves the challenge of multi-tasking cross-validation through the knowledge resources shared by the meta-learning tasks. It implements the security aggregation mechanism to avoid the additional communication overhead the committee strategy may cause.

To solve the above problems, based on the fault diagnosis method of artificial intelligence, this paper proposes a multi-task causal knowledge fault diagnosis method of PMSM-ITSF based on ML. The method first analyzes PMSM-ITSF and the three-phase current, positive-sequence current (PSC), negative-sequence current (NSC), zero-sequence current (ZSC), and electromagnetic torque (EMT). The motor’s fault characteristic quantity under different fault degrees is selected. Secondly, Simulink, Simplorer, and Maxwell were used to build a simulation platform to obtain experimental data. Thirdly, the dataset is introduced into the learning network for training, and multi-task learning (MTL) diagnosis of PMSM-ITSF fault degree and location is realized under small samples. Then, a cross-domain experimental environment is created to test and verify. Finally, the PMSM-ITSF fault knowledge graph is constructed and stored in the Neo4j database. This helps to assess the extent of the fault quickly, locate the fault location, and query the cause of the fault. It also effectively eliminates the false fault caused by noise and other factors, which provides strong knowledge decisions for the rapid response and accurate repair of industrial robot motor faults.

The remaining sections of this article are arranged as follows. Section 2 introduces PMSM-ITSF, selecting feature quantities for fault diagnosis. Section 3 introduces the network type, structure, and knowledge base of PMSM-ITSF multi-task causal knowledge fault diagnosis based on ML. In Section 4, a joint simulation platform of PMSM-ITSF is constructed, and the dataset obtained by the joint simulation is input into the proposed algorithm for training to identify and diagnose different fault states and lock fault locations. In Section 5, experimental results are summarized to demonstrate the effectiveness of the proposed algorithm.

## 2. Materials and Methods

**Figure 1:**
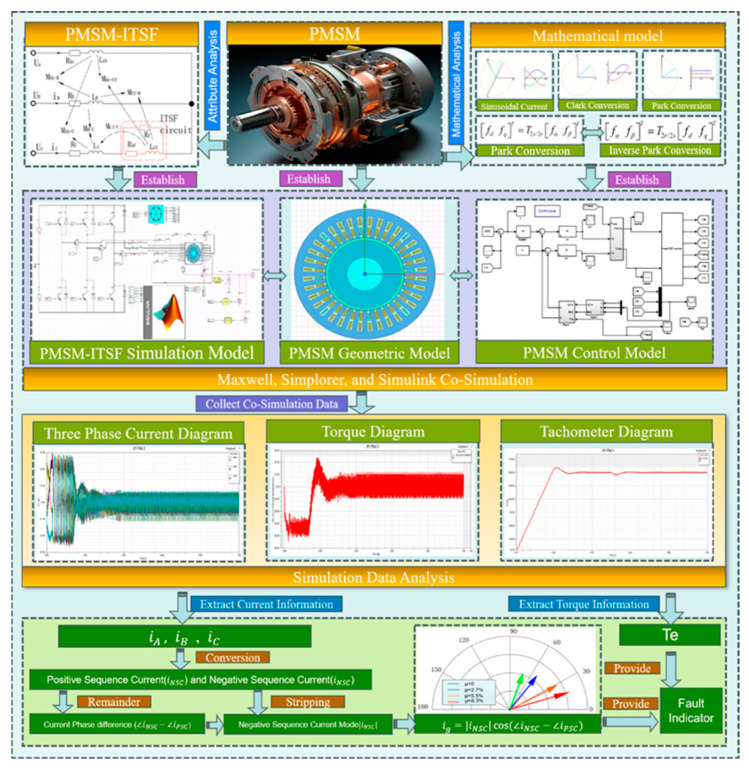
PMSM-ITSF Simulation Model.

Attribute analysis and mathematical analysis were carried out on PMSM-ITSF, and the simulation model of ITSF was established, as shown in Figure 1.

### 2.1. PMSM Mathematical Model

PMSM has the properties of being multi-variable, nonlinear, strong coupling, etc. In order to better study PMSM-ITSF, the following assumptions are made. (1) Ignore the core saturation of PMSM, ignoring the influence of eddy current, hysteresis loss, and slot torque. (2) The conductivity of the magnetic material and the damping winding on the rotor are zero. (3) The waveform of the induced potential is sine. In the ABC three-phase stationary coordinate system, assuming that the three-phase current passing through the coil is symmetrical and has the same amplitude, the voltage equation of PMSM is expressed, as shown in Equation (1).

(1) $$\left\{\begin{array}{c} u_{A}=R_{S}i_{A}+\frac{d\varphi_{A}}{dt}\\ u_{B}=R_{S}i_{B}+\frac{d\varphi_{B}}{dt}\\ u_{C}=R_{S}i_{C}+\frac{d\varphi_{C}}{dt} \end{array}\right.$$

where $u_{A}$, $u_{B}$, $u_{C}$ is the phase voltage of the ABC phase; $R_{S}$ is the phase resistance of the stator winding; $\varphi_{A}$, $\varphi_{B}$, $\varphi_{C}$ is the flux of the stator winding.

The PMSM studied uses star connections, the electrical angle difference is $\varphi_{f}$, and the current sum is 0. The flux equation in natural coordinate system, as shown in Equation (2):

(2) $$\left\{\begin{array}{c} \varphi_{A}=L_{AA}i_{A}+M_{AB}i_{B}+M_{AC}i_{C}+\psi_{f}\cos\left(\theta \right)\\ \varphi_{B}=L_{BB}i_{B}+M_{BA}i_{A}+M_{BC}i_{C}+\psi_{f}\cos\left(\theta -120^{{}^{\circ}}\right)\\ \varphi_{C}=L_{CC}i_{C}+M_{CA}i_{A}+M_{CB}i_{B}+\psi_{f}\cos\left(\theta +120^{{}^{\circ}}\right) \end{array}\right.$$

where $i_{A}$, $i_{B}$, $i_{C}$ is the three-phase current between the stator windings; $\varphi_{A}$, $\varphi_{B}$, $\varphi_{C}$ is the flux linkage of the stator winding; L is inductance between stator windings; M is mutual inductance between stator windings; $\psi_{f}$ is the flux linkage amplitude of the permanent magnet; θ is the rotor electrical angle.

When PMSM converts electrical energy into mechanical kinetic energy, electromagnetic torque (EMT), as shown in Equation (3):

(3) $$T_{e}=\frac{1}{2}n_{p}\frac{\partial }{\partial \theta_{m}}\left({\left[\begin{array}{c} i_{A}\\ i_{B}\\ i_{C} \end{array}\right]}^{T}\times \left[\begin{array}{c} \varphi_{A}\\ \varphi_{B}\\ \varphi_{C} \end{array}\right]\right)$$

where $n_{p}$ are the number of magnetic poles, $\theta_{m}=\theta /n_{p}$ is the mechanical angle position, and the current and flux matrix multiplication, which represent magnetic energy storage.

PMSM runtime balance equation, as shown in Equation (4):

(4) $$T_{e}-T_{L}=J\frac{d\omega_{m}}{dt}+B\omega_{m}$$

where $T_{L}$ is the torque of the mechanical load, $\omega_{m}$ represents the mechanical angular velocity, J is the moment of inertia, B is the damping coefficient.

### 2.2. ITSF Model

**Figure 2:**
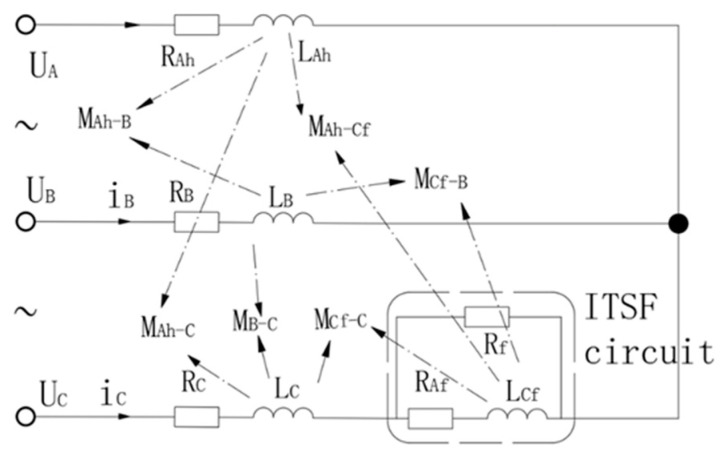
PMSM-ITSF Schematic Drawing.

ITSF occurs when the insulation layer between the two windings of the motor’s stator is damaged, causing a short circuit fault. The short circuit generates a short circuit current $i_{f}$, and the remaining insulation layer between the adjacent turns is destroyed, which leads to the aggravation of the fault, causes the three-phase current imbalance of the motor, and affects the motor’s regular operation. The schematic diagram of ITSF is shown in Figure 2.

The total number of phase C coils is *n*; each coil contains N winding turns. Assume that the phase C short circuit fault is shorted by the short circuit resistor $R_{f}$, and the short circuit current generated is $i_{f}$, the number of short turns is $N_{f}\left(N_{f}<nN\right)$, the number of turns is shown in Equation (5):

(5) $$\eta =N_{f}/nN$$

The ITSF turns ratio directly determines the degree of motor failure. It can be seen from the ITSF model diagram that when ITSF occurs in the C-phase winding of the PMSM motor, the stator winding of the C-phase:

(6) $$R=R_{AS}+R_{S}$$

The short circuit turns ratio is η, then:

(7) $$R_{S}=\eta R$$

(8) $$\left\{\begin{array}{c} L_{As}=\eta^{2}L_{AA}\\ L_{Ah}={\left(1-\eta \right)}^{2}L_{AA}\\ M_{As-B}=\eta M_{AB}\\ M_{As-C}=\eta M_{AC}\\ M_{Ah-B}=\left(1-\eta \right)M_{AB}\\ M_{Ah-C}=\left(1-\eta \right)M_{AC}\\ M_{Ah-Af}=\eta \left(1-\eta \right)M_{AC} \end{array}\right.$$

At this time, the voltage–current relationship of PMSM-ITSF is:

(9) $$\left[\begin{array}{c} u_{A}-u_{n}\\ u_{B}-u_{n}\\ u_{C}-u_{n}\\ 0 \end{array}\right]=\left[\begin{array}{cccc} R & 0 & 0 & -\eta R\\ 0 & 0 & 0 & 0\\ 0 & 0 & 0 & 0\\ \eta R & 0 & 0 & -\eta R-R_{f} \end{array}\right]\left[\begin{array}{c} i_{A}\\ i_{B}\\ i_{C}\\ i_{f} \end{array}\right]+\left[\begin{array}{cccc} L_{AA} & M_{AB} & M_{AC} & \eta L_{AA}\\ M_{AB} & L_{AA} & M_{BC} & \eta M_{AB}\\ M_{AC} & M_{BC} & L_{AA} & \eta M_{AC}\\ \eta L_{AA} & \eta M_{AB} & M_{AC} & \eta^{2}L_{AA} \end{array}\right]\frac{d}{dt}\left[\begin{array}{c} i_{A}\\ i_{B}\\ i_{C}\\ i_{f} \end{array}\right]+\frac{d}{dt}\left[\begin{array}{c} \varphi_{A}\\ \varphi_{B}\\ \varphi_{C}\\ \varphi_{f} \end{array}\right]$$

where $u_{n}$ is the neutral voltage. Easy to get, short circuit current is expressed as:

(10) $$i_{f}=\frac{\eta (u_{A}-u_{n})}{\eta (1-\eta )R+R_{s}}$$

Therefore, the fault state of PMSM-ITSF can be effectively identified by comparing the electrical signal changes caused by PMSM-ITSF before and after. However, in the actual operation of the motor, the unbalanced supply voltage (USV) caused by the external environment will occur within the allowable range, which is similar to the current signal of the stator ITSF. It is easy to false events, and it is difficult to distinguish two kinds of faults by fault current. The motor fault will affect the phase unbalance of PMSM, and the negative sequence current is one of the important indexes of PMSM phase unbalance. The relationship between PMSM’s three-phase current and positive sequence current (PSC), negative sequence current (NSC), and zero sequence current is shown in Equation (11):

(11) $$\left[\begin{array}{c} i_{+}\\ i_{-}\\ i_{0} \end{array}\right]=\frac{1}{3}\left[\begin{array}{ccc} 1 & e^{j120^{\circ }} & e^{2j120^{\circ }}\\ 1 & e^{2j120^{\circ }} & e^{j120^{\circ }}\\ 1 & 1 & 1 \end{array}\right]\left[\begin{array}{c} i_{A}\\ i_{B}\\ i_{C} \end{array}\right]$$

when $a=e^{j120^{\circ }}$, the positive sequence current of PMSM can be expressed as:

(12) $$i_{+}=\frac{\left(i_{A}+ai_{B}+ai_{C}^{2}\right)}{3}$$

The negative sequence current of PMSM can be expressed as:

(13) $$i_{-}=\frac{\left(i_{A}+ai_{B}^{2}+ai_{C}\right)}{3}$$

To diagnose mild ITSF and ITSF levels under various conditions, with the increase in ITSF, the phase difference between PSC and NSC decreases, and the amplitude of NSC increases. Therefore, the magnitude of the NSC can be multiplied by the phase difference between the PSC and the NSC as a fault indicator [42], which can be obtained:

(14) $$i_{g}=|i_{Nsc}|\cos(∠i_{Nsc}-∠i_{Psc})$$

The data collected by the current sensor are the instantaneous current value of the motor; instantaneous value and practical value can be converted to each other, as shown in Equation (15):

(15) $$I=\sqrt{\frac{1}{T}\int_{0}^{T}i^{2}(t)}$$

In the motor’s actual operation, the change of the three-phase current and its positive and negative sequence current caused by the load and environmental change under non-ITSF makes it easy to cause false misjudgment, and it is not easy to judge the motor’s state accurately and effectively. To improve the accuracy of motor ITSF diagnosis, EMT is used to compensate for this defect. As a core index of electromagnetic characteristics of PMSM, EMT is a rotating torque generated by the interaction between the current in the motor rotor and the magnetic flux of each magnetic pole in the rotating magnetic field. This parameter not only profoundly affects PMSM’s safety performance but also directly relates to the motor’s service life and working efficiency, which is an indispensable key element for evaluating and optimizing the motor’s comprehensive performance.

The input power of PMSM is shown in Equation (16):

(16) $$P_{1}={\mathrm{mUI}}_{1}\cos\varphi ={\mathrm{mUI}}_{1}\cos(\psi -\theta )=m({\mathrm{UI}}_{d}\cos\theta +{\mathrm{UI}}_{q}\cos\theta )$$

where *θ* is the power angle, representing the lead of the voltage vector relative to the no-load electromotive force, that is $\psi =\arctan(I_{d}/I_{q})$; $I_{d}$ and $I_{q}$ are the direct axis and quadrature axis components of stator current, respectively. On the premise of ignoring the resistance loss of the stator winding, according to the voltage–current equation of PMSM, $I_{d}$ and $I_{q}$ of PMSM are derived so that PMSM-EMT can be expressed as shown in Equation (17):

(17) $$T_{em}=\frac{P_{em}}{\Omega }=\frac{P_{em}P}{\omega }\approx \frac{mpUE_{0}}{\omega X_{d}}\sin\theta +\frac{mpU^{2}}{2\omega }(\frac{1}{X_{q}}-\frac{1}{X_{d}})\sin2\theta$$

where Ω is the mechanical angular speed of the motor; ω is the electric angular speed of the motor.

PMSM-EMT consists of two cores: magnetoresistive torque and permanent magnet torque. The difference between the axial and axial magnetoresistive causes the magnetoresistive torque. The permanent magnet torque is the result of the interaction between the magnetic field caused by the reaction between the magnetic field of the permanent magnet and the armature.

When the PMSM uses a surface mount rotor assembly, the reluctance torque will not exist. It is worth noting that the electromagnetic torque of PMSM is closely related to the magnetic field strength of PMSM and the winding current [43]. Under normal operating conditions, EMT will fluctuate regularly within a particular threshold value. However, when PMSM-ITSF occurs, the fluctuation amplitude of EMT will rapidly increase. It will increase rapidly with the increase of short circuit turns, which will deviate from the typical trajectory. Therefore, the EMT feature quantity can effectively improve fault diagnosis accuracy based on the fault indicator.

## 3. Causal Knowledge Fault Diagnosis for Multi-Task ML

**Figure 3:**
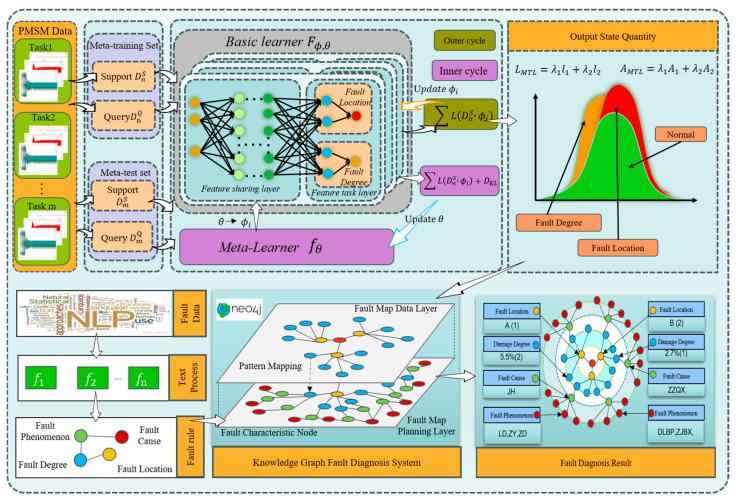
Multi-task meta-learning PMSM-ITSF causal knowledge diagnosis framework.

Figure 3 shows the multi-task causal knowledge fault diagnosis model framework of PMSM-ITSF based on ML proposed in this paper. The framework consists of a multi-task fault diagnosis module based on ML and a causal knowledge-building module based on industrial robots’ PMSM-ITSF. In the causal knowledge-building module based on industrial robots’ PMSM-ITSF, we focus on in-depth analysis and information extraction of complex relationships between industrial robots’ PMSM-ITSF, aiming to build an accurate industrial robots’ PMSM-ITSF rule chain and provide a solid theoretical foundation for subsequent fault diagnosis. The multi-task fault diagnosis module of PMSM-ITSF based on ML makes full use of the advantages of ML so that the model can accurately and efficiently diagnose different fault locations and damage degrees of industrial robots’ PMSM-ITSF in the context of small samples. This algorithm shows excellent learning ability and performs well in generalization, providing strong technical support for PMSM-ITSF fault diagnosis.

By closely integrating advanced diagnosis algorithms with detailed fault rule chains, we can quickly and accurately deduce key diagnostic results such as “Fault Degree”, “Fault Location”, and “Fault Cause” of industrial robots’ PMSM, provide similar fault list queries, provide auxiliary decision-making for fault diagnosis, and effectively eliminate false events.

### 3.1. Multi-Task Learning Network

**Figure 4:**
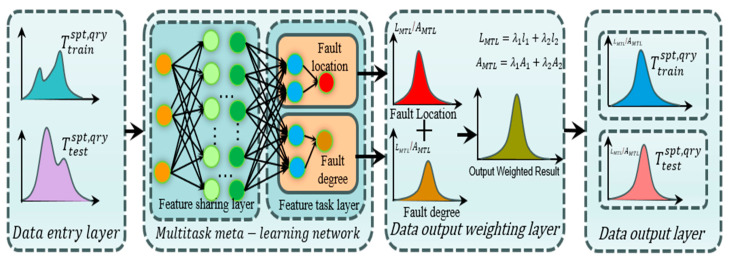
Multi-task learning network.

As shown in Figure 4, multi-task learning (MTL) with one input and two outputs is given; on the left side of the figure are MTL dataset inputs, and on the right side are MTL dataset outputs, each corresponding to the corresponding task. The network is mainly composed of a feature-sharing layer and a feature-task layer. The task-sharing layer aims to learn rich task-sharing features from input signals, thus mining the correlation between ITSF degree and location tasks. Similar task-sharing parameters can improve performance, while different tasks will introduce noise reduction performance [44]. Having shared and exclusive parameters mitigates this noise and improves performance for each task.

The fault location and fault degree diagnosis tasks of PMSM-ITSF are multi-label classification tasks. It can be optimized by the cross-entropy loss function, which is calculated as:

(18) $$l_{1}=\sum_{i=1}^{N}\left(y_{i}\cdot {\mathrm{logp}}_{i}+\left(1-y_{i}\right)\cdot \log\left(1-p_{i}\right)\right)$$

where $y_{i}$ and $p_{i}$ represent expectation and prediction probability, respectively, and *N* represents the number of training samples.

Therefore, the loss function of MTL proposed in this paper can be obtained by combining the loss functions of the above two tasks. Considering the differences in data distribution and the importance of PMSM-ITSF fault location and fault degree tasks, the MTL loss value is calculated as shown in Equation (19):

(19) $$L_{MTL}=\lambda_{1}l_{1}+\lambda_{2}l_{2}$$

Similarly, the MTL accuracy formula, as shown in Equation (20):

(20) $$A_{MTL}=\lambda_{1}A_{1}+\lambda_{2}A_{2}$$

where $L_{MTL}$ multi-task learning model loss; $A_{MTL}$ multi-task learning model accuracyy, $\lambda_{1}$ and $\lambda_{2}$ are balance factors, which are 0.5.

### 3.2. Meta-Learning Training Process

Meta-learning (ML) methods can be divided into optimization-based methods and metric-based methods. Metric-based methods belong to non-parameterization categories, such as twin networks, relational networks, matching networks, and other network models. Optimization-based methods belong to the parametric method, and one of the typical representatives is model-agnostic meta-learning (MAML). The diagnosis method based on MAML uses multiple fault classification tasks with known working conditions to train the model and can automatically learn the optimal initialization parameters of the model through a gradient descent strategy, thus obtaining prior fault knowledge. When faced with a new small sample test task, MAML will migrate these initial parameters and apply them to a new environment, fine-adjusting these parameters to improve the adjusted model’s generalization ability so that the model can show excellent performance on the new target task [45].

**Figure 5:**
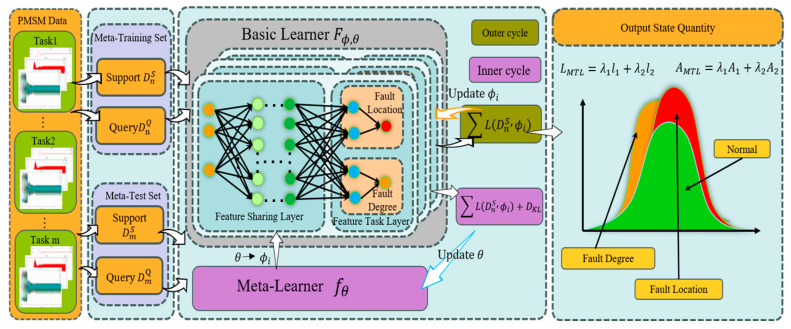
Meta-learning training process.

In the framework of MAML, the combination of the basic learner and the meta-learner, as shown in Figure 5, can automatically learn the model’s optimal initialization parameters. When faced with a new test task, MAML migrates these initial parameters and applies them to a new environment, fine-tuning them through gradient descent strategies to ensure the model performs well on the new task.

Each task contains a training set (a support set and a query set) and a test set; usually, according to the different forms of training tasks, the support set and query set of each task are set in the form of N-way, K-shot, and N-way, Q-query, respectively. The test dataset is divided into N-way, Q-query. The query set optimizes the model, and the test set fine-tunes the model parameters. In model training, the support set is used to train the model parameters, which is applied to the model of MTL fault location and fault degree as the core data for training, and the preliminary model parameter $\theta^{\prime }$ is obtained. Then, the query set takes on the task of optimizing parameter $\theta^{\prime }$ and fine-tuning it to obtain a better model parameter θ. In this process, the basic learner is mainly used to dig deeply into the unique characteristics of each task. At the same time, the meta-learner is dedicated to capturing the commonalities between tasks and realizing the effective transfer and integration of knowledge.

Enter the meta-testing phase, using the test set to fine-tune the initial parameters θ, aiming to evaluate and improve the generalization ability of the model to ensure that the model can perform well in the new task environment scenarios. Through such a strategy, we can not only train the model parameter θ that performs better in the new task environment but also ensure high precision on the training dataset, effectively avoid the risk of overfitting on the test dataset, and realize the dual improvement of model performance and generalization ability.

**Figure 6:**
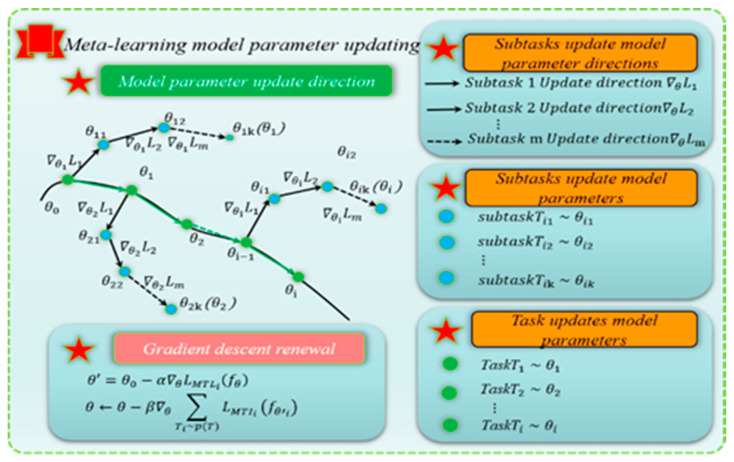
MAML stochastic gradient descent algorithm.

The training process of the inner and outer layers of MAML model parameters is shown in Figure 6. In the inner loop structure, the parameter updating process of MAML is as shown in Equation (21):

(21) $$\theta^{\prime }=\theta_{0}-\alpha \nabla_{\theta }L_{MTL_{i}}(f_{\theta })$$

where θ is the optimal parameter updated on this task; $T_{i}$ is the i-th task in the task set. α is the learning rate of the inner loop network; $f_{\theta }$ is the inner loop network structure of parameter θ; $L_{MTL_{i}}$ is the loss of a specific task; $\nabla_{\theta }L_{MTL_{i}}(f_{\theta })$ is the loss gradient.

In the outer loop structure, MAML requires adapting quickly to new tasks. Therefore, the optimal parameter θ is required to have the most minor loss error for all different tasks in the following Equation (22):

(22) $$\min_{\theta }\sum_{T_{i}-p(T)}L_{MTL_{i}}(f_{\theta^{\prime }})=\sum_{T_{i}-p(T)}L_{MTL_{i}}(\theta^{\prime }-\alpha \nabla_{\theta }\sum_{T_{i}-p(T)}L_{MTL_{i}}(f_{\theta }))$$

where p(T) is the distribution of task sets. Finally, MAML can update parameters by random gradient descent, and the process is as shown in Equation (23):

(23) $$\theta \leftarrow \theta -\beta \nabla_{\theta }\sum_{T_{i}-p(T)}L_{MTL_{i}}(f_{\theta_{i}^{\prime }})$$

where β is the learning rate of the meta-learner.

Through the above description, the pseudo-code of the algorithm in this paper is shown in Algorithm 1.

The pseudo-code is as follows.

**Algorithm 1** A Model-Agnostic Multi-Task Meta-Learning Algorithm
Input: x_spt ($i_{A}$,$i_{B}$*,*$i_{C}i_{g}$*,*$T_{e}$)*,* x_qry ($i_{A}$,$i_{B}$*,*$i_{C}i_{g}$*,*$T_{e}$)

Output: y_spt_deg(Deg), y_spt_loc(*Loc*)

Require: p(T): distribution over tasks p(T)

Require: α,β: step size hyperparameters (inner loop α, outer loop β)

1: Initialize model parameters θ (weights of Multi-Task Network)

2: while not done do

3:   Sample batch of tasks $T_{i}$~p(T)

4:   for all tasks $T_{i}$ in the batch do

5:     Sample K datapoints $D=\{x^{\left(j\right)},y^{\left(j\right)}\}$ from $T_{i}$ (support set)

6:     x_spt, y_spt_deg, y_spt_loc← subset of x_spts, y_spts_deg, y_spts_loc corresponding to task $T_{i}$

7:     Evaluate $\nabla_{\theta }L_{T_{i}}(f_{\theta })$ using $D\;and\;L_{T_{i}}$($L_{T_{i}}={0.5*(L}_{sec\_T_{i}}+L_{loc\_T_{i}})$ in Equation (2) or Equation (3)

8:     Compute gradients of loss w.r.t. θ on support set (x_spt_i, y_spt_i)

9:     Compute adapted parameters with gradient descent: $\phi^{T_{i}}=\theta -{\alpha \nabla }_{\theta }L_{T_{i}}(f_{\theta })$

10:      Sample K datapoints $D_{i}^{\prime }=\{x^{\left(j\right)},y^{\left(j\right)}\}$ from $T_{i}$ for the meta-update (query set)

11:      x_qr, y_qry_deg, y_qry_loc←subset of x_querys, y_qrys_deg, y_qrys_loc corresponding to task $T_{i}$

12:      Evaluate model with adapted parameters $\phi^{T_{i}}$ on query set $D_{i}^{\prime }$

13:      Compute loss $L_{T_{i}}\left(f_{\phi^{T}i}\right)={0.5*(L}_{deg\_\phi^{T}i}+L_{loc\_\phi^{T}i})$ on query set (x_qry_i, y_qry_i)

14:      Evaluate $\nabla_{\theta }L_{T_{i}}(f_{\theta })$ using $D\;and\;L_{T_{i}}$($L_{T_{i}}={0.5*(L}_{deg\_T_{i}}+L_{loc\_T_{i}})$)

15:      Compute gradients of loss w.r.t. θ on support set (x_spt_i, y_spt_i)

16:      Compute adapted parameters with gradient descent: $\phi^{T_{i}}=\theta -{\alpha \nabla }_{\theta }L_{T_{i}}(f_{\theta })$

17:      Sample K datapoints $D_{i}^{\prime }=\{x^{\left(j\right)},y^{\left(j\right)}\}$ from $T_{i}$ for the meta-update (query set)

18:      x_qry_i, y_qry_deg_i, y_qry_loc_i←subset of y_qrys_deg_i, y_qrys_loc_i corresponding to task $T_{i}$

19:      Evaluate model with adapted parameters $\phi^{T_{i}}$ on query set $D_{i}^{\prime }$

20:      Compute loss $L_{T_{i}}\left(f_{\phi^{T}i}\right)={0.5*(L}_{deg\_\phi^{T}i}+L_{loc\_\phi^{T}i})$ on query set (x_qry_i, y_qry_i)

21:      Accumulate validation accuracy (Acc = ${0.5*(A}_{deg\_val\_i}+A_{loc\_val\_i})$)

22:    Accumulate gradients w.r.t. θ for meta-update

23:    end for

24:    Update $\theta \leftarrow \theta -\beta \nabla_{\theta }\sum_{T_{i}∼p\left(T\right)}\;L_{T_{i}}\left(f_{\phi^{T}i}\right)$ using each $D_{i}^{\prime }$ and $L_{deg\_\phi^{T}i},L_{loc\_\phi^{T}i}$ in Equation (2) or Equation (3)

25: end while


### 3.3. Knowledge Graph Construction Method Based on PMSM-ITSF

Knowledge graph (KG) is a technology that finds entities and relationships in information and builds a structured semantic network knowledge base with nodes representing entities and edges representing relationships to provide solutions for causal reasoning of faults [46].

In fault diagnosis, KG technology establishes a causal knowledge base based on knowledge and relationships in related fields. This knowledge base can help maintenance personnel quickly and effectively locate and evaluate faults and make decisions about maintenance programs [47]. The KG is more common in fault diagnosis applications in medicine [48], intelligent manufacturing [49], and other fields. For example, reference [50] summarized the commonly used databases of knowledge embedding models in drug discovery. Reference [51] used knowledge graph technology to identify symptoms from Chinese HER data and make treatment decisions. Reference [52] used KG technology to construct electrical equipment fault KG through the text information of electrical equipment operation and maintenance, visualizing the correlation between the faulty equipment and components. In order to improve the extraction efficiency of text information, reference [53] used DL to extract equipment fault information and build a KG, showing relatively efficient construction ability.

Aiming at the problem that the traditional PMSM fault diagnosis method lacks the ability of fault information structured management, a method of constructing a fault information KG based on PMSM is proposed. Its data are divided to adapt to ML training. Firstly, the fault rule chain of the KG is extracted from PMSM-ITSF text data. Secondly, fault characteristic information is extracted from the equipment operation data, and corresponding labels are made. Finally, the fault characteristic information is combined with the fault rule chain to generate node information in the form of a unified description of “Fault location”, “Fault degree”, “Fault phenomenon”, and “Fault cause” to complete the construction of the KG.

The definition formula of the PMSM fault information KG as shown in Equation (24):

(24) G=(E,P,S,R)

where $E=\left\{e_{1},e_{2},\ldots e_{E}\right\}$ represents a collection of entities, e is the most basic element of the KG. $R=\left\{r_{1},r_{2},\ldots ,r_{R}\right\}$ represents a set of relationships, where r is an edge in the KG that represents a specific connection between different entities. $F=\left\{f_{1},f_{2},\ldots ,f_{F}\right\}$ represents a set of facts, and has (h,r,t)∈f, thus obtaining the definition of triples in the KG: f={(h,r,t)|h,t∈E,r∈R}, where *h* represents the head entity, *r* represents the relationship, and *t* represents the tail entity.

**Figure 7:**
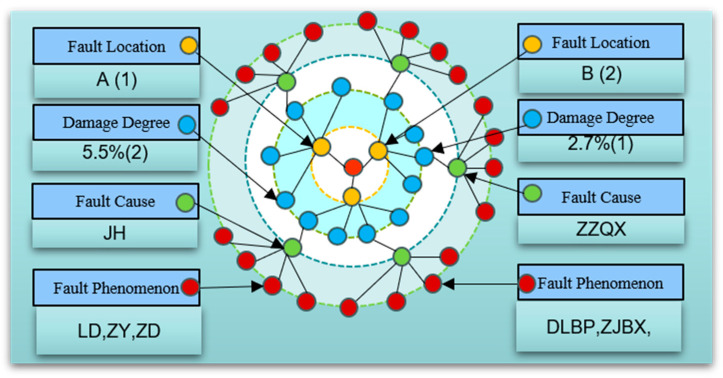
Knowledge graph fault diagnosis.

**Figure 8:**
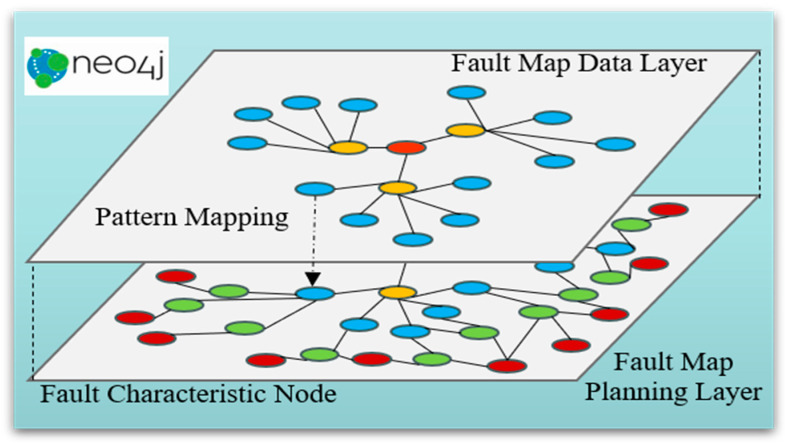
Knowledge graph fault diagnosis system.

The fault text node is the entity expression under a specific working condition, and the fault data node is the entity expression of the sampling fragment of the PMSM running signal under multiple working conditions. As shown in Figure 7, the text is extracted as the fault rule method, taking the PMSM operation record file under a certain working condition as an example. The characteristic signal labels of “Fault location”, “ Fault degree”, and “Fault phenomenon” are combined with the fault text nodes of “Fault location”, “damage depth”, and “Fault manifestation” in the triplet relationship of <Fault data node, belong to, Fault text node>, and the “fault cause” is derived through the KG rule chain, as shown in Figure 8.

A combination of Python (3.9.7) and Neo4j (1.5.9) technologies was chosen to build and store KG for PMSM-ITSF. Neo4j, an excellent NoSQL graph database, is unique because the data are stored in a networked form rather than a traditional table structure. It is a high-performance graphics storage solution and a powerful built-in graphics computing power engine. In the Neo4j graph database, the CREATE() statement is used to create nodes and assign labels and attributes to these nodes to describe their characteristics accurately. By combining the MATCH() and CREATE() statements, you can quickly establish relationships between two nodes and assign attributes to those relationships. When all the elements are created and connected, a complex knowledge network structure emerges and is stored in the Neo4j graph database as a graph structure, which provides strong support for subsequent fault analysis, prediction, and decision-making.

For the loading, searching, matching, and sorting operations of entities, relationships, attributes, and other elements, Neo4j provides a wealth of query language support and intuitively shows the association and rule chain between data through the graphical structure so that the knowledge hidden in the data relationship can be mined and utilized. When querying and analyzing PMSM-ITSF, the RETURN() statement is used to extract the required data, the WHERE() statement to set the query criteria, and the MATCH() statement to locate a specific node or relationship. These functions together constitute Neo4j’s powerful query and analysis capabilities.

## 4. Analysis of Experimental Results

Due to the complexity of the PMSM working environment, many factors interfere with the judgment of the motor state, leading to a lack of sample data and poor data quality in PMSM fault diagnosis. The characteristic quantities used in this paper are the three-phase current of PMSM, its positive and negative sequence current, and electromagnetic torque, which are used as the inputs of the learning network model.

The parameters of the PMSM motor selected in this paper are shown in Table 1 after consulting the motor information and combining the actual situation.

### 4.1. PMSM Model and Fault Feature Collection

In order to verify the effectiveness of the proposed method, limited by the experimental conditions of the physical entity motor, this paper simulates the experimental dataset by building a mathematical model. In this paper, MATLAB/Simulink (2018), Simplorer (2017), and Maxwell (2017) were used for joint simulation, as shown in Figure 1. Simulate PMSM-ITSF to conduct experiments under different fault states, obtain three-phase current amplitude, positive and negative sequence current, electromagnetic torque, and other characteristic components under corresponding motor states, and indirectly obtain fault indicators. In addition, three-phase current amplitude, fault indicator, and electromagnetic torque are inputs for the learning network model, and the output is set as fault degree and location. Status label corresponding to the motor sample dataset. Since the probability of two-phase or more faults co-occurring during the operation of PMSM is minimal, this paper only studies single-phase faults. The sample labels of motors set in the ITSF sample dataset are shown in Table 2, with a total of 12 combinations, but the health state is classified as 1, with a total of 10 state combinations.

### 4.2. Experimental Settings

All the methods in this paper are implemented on the computer, the hardware environment of which is GPU RTX3060 and CPU core i7. In addition, all models are built on Pytorch 1.8.1 and CUDA 10.2. The experimental data in this paper are as follows. When PMSM runs for 0.5 s, load 3 N and 5 N are applied, respectively, under different fault degrees and positions, and data are collected after the motor runs smoothly. A total of 1200 samples of evenly distributed data are collected and normalized, among which some fault samples are shown in Table 3. The ratio of the training set and test set is 7:3.

### 4.3. Experimental Test and Result Analysis

**Figure 9:**
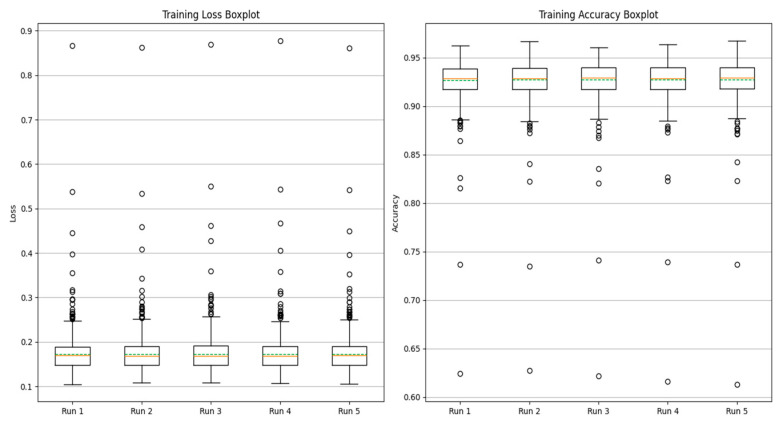
Loss value and accurate value of the number of internal and external cycles.

As shown in Figure 9, the number of inner and outer layer loop iterations on the experimental data with the method proposed in this paper shows that the loss value is lower, and the accuracy is better when 5 times of inner and outer layer loop iterations are performed for each task. Therefore, the experiments in this paper are carried out by 5 times loop iterations.

All the experimental results in this paper were averaged over five experiments to reduce the influence of random errors. In the ablation experiment, SGD was used in the inner layer, and Adam was used in the outer layer. The learning rate of the inner and outer layer cycles was 0.001, and the training results of the inner and outer layer cycles for each task were good.

A comparative experiment on the fault degree of PMSN-ITST was conducted to better divide task data in the support set, and the results are shown in Table 4.

In order to obtain a better task data division of the query set, a comparative experiment was conducted on the fault degree of PMSN-ITST, and the results are shown in Table 5.

As shown in Table 4 and Table 5 above, under the training mode of 4-way, 5-shot support set and 4-way, 5-query set, ML achieves the best diagnostic accuracy, which is stable at 99.75% ± 0.25%. As shown in Table 6, compared with GAN + SAE with 16,500 datasets, the increase is 0.35%; compared with Cycle GAN-DAE with 6000 datasets, the increase is 0.91%; compared with GAN with 1200 datasets, the increase is 9.75%.

**Figure 10:**
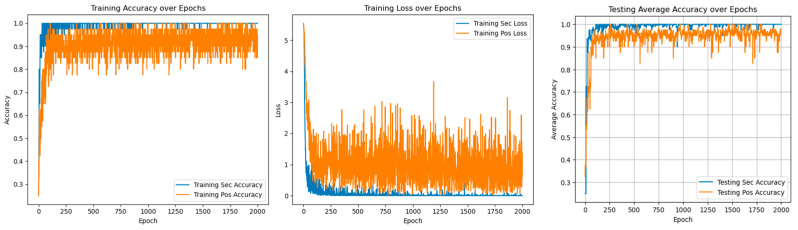
Fault diagnosis result of single-task unit learning.

**Figure 11:**
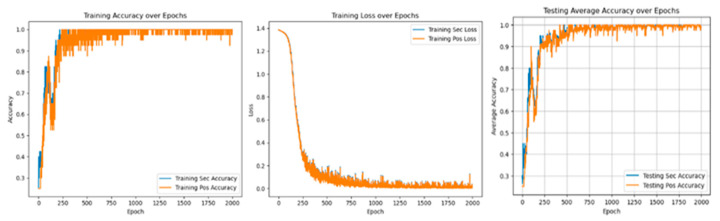
Fault diagnosis results of multi-task meta-learning.

In multi-task learning, sharing parameters of similar tasks can improve performance, while not sharing parameters of similar tasks will introduce noise reduction performance. At the same time, sharing and unique parameters can alleviate such noise, as shown in Figure 10 and Figure 11. 

Table 7 shows that in the training and verification stages, the accuracy of fault degree and fault location are both stable at 98.53 ± 0.35% and 94.39 ± 0.83% under single-task learning. However, in the diagnosis of fault location, the loss value and accuracy of single-task learning are lower than those of multi-task learning, and there are large fluctuations. The result shows that introducing the multi-task network can improve diagnostic accuracy.

In order to better verify the feasibility of the method, the best data partitioning method of this method is screened out when the inner and outer layers are iterated 5 times for each task, as shown in Table 8. The fault degree and fault location diagnosis accuracy of each method in PMSM-ITST are shown below.

**Figure 12:**
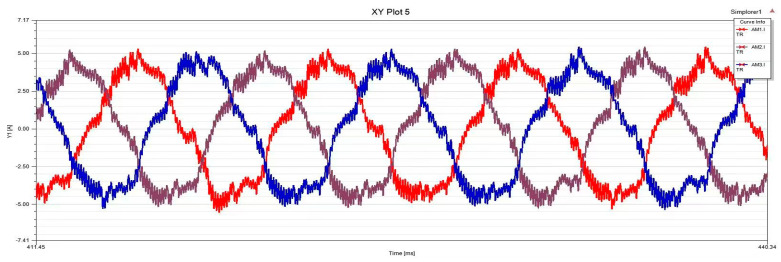
Voltage balance, Normal.

**Figure 13:**
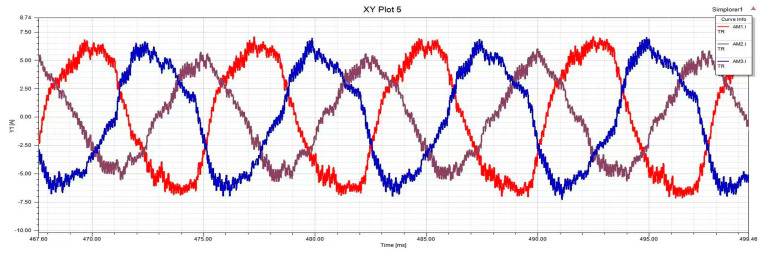
Voltage balance, Phase C minor inter-turn short circuit fault.

**Figure 14:**
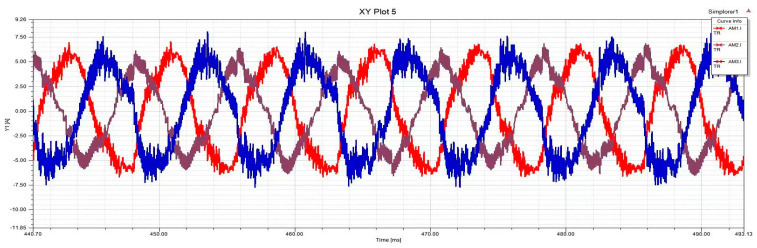
The voltage unbalance is 0.56%, Phase C minor inter-turn short circuit fault.

**Figure 15:**
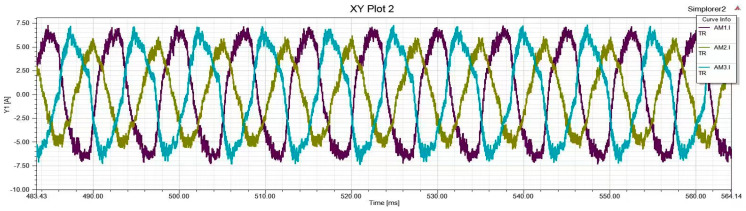
The voltage unbalance is 2.8%, Phase C minor inter-turn short circuit fault.

In order to achieve better generalization performance of the proposed method model, it is suitable for cross-domain fault diagnosis under more severe conditions. In this paper, ITSF fault diagnosis is used to conduct experiments under various voltage unbalanced degrees; the horizontal coordinate is ms, and the vertical coordinate is A, as shown in Figure 12, Figure 13, Figure 14 and Figure 15. Because in the actual operation of the motor, the unbalanced supply voltage (USV) will also occur within the allowable range of the stator ITSF caused by the current signal changes, so different cases of different voltage unbalanced degree ITSF will be studied. According to the “GB/T 15543-2008 Power quality three-phase voltage imbalance”, the negative sequence voltage imbalance should not exceed 2%, and the short-term should not exceed 4% [56]. The calculation formula is as follows:

(25) $$\left\{\begin{array}{c} U_{AC}=(1+\delta )\times 220\sqrt{2}\sin(2\pi ft)\\ U_{AB}=220\sqrt{2}\sin(2\pi ft-\frac{2\pi }{3})\\ U_{BC}=-U_{AC}-U_{AB} \end{array}\right.$$

The above is the calculation formula for the voltage unbalance degree. When δ=0.01, the voltage unbalance degree is 0.56%, and when δ=0.05, it is 2.8%. This paper divides the task training set into 4-way and 5-shot support sets and 4-way and 5-query sets, which are used to carry out relevant experiments of different voltage unbalance degrees under the conditions of 3 N·m and 5 N·m motor loads.

**Figure 16:**
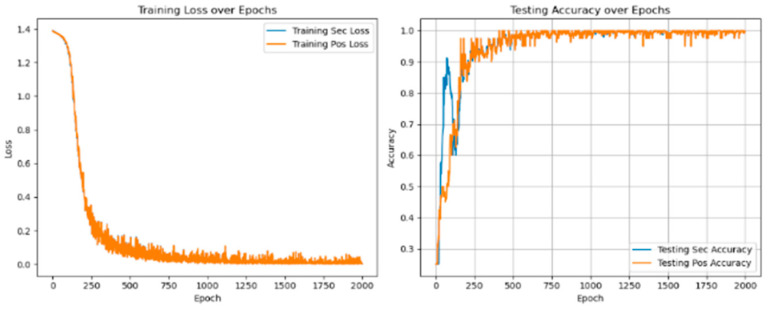
Voltage unbalance 0.56% diagnostic loss value and accurate value.

**Figure 17:**
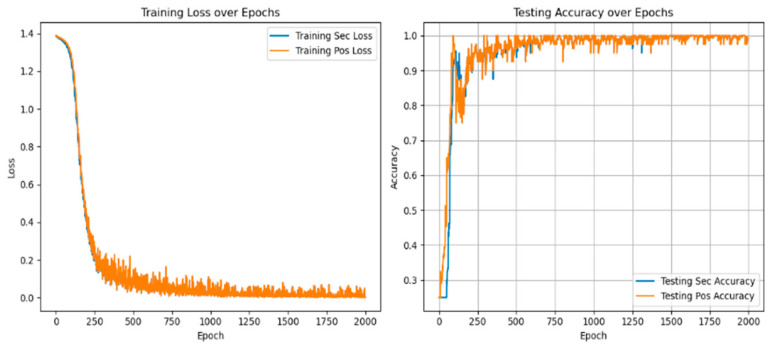
Voltage unbalance 2.8% diagnostic loss value and accurate value.

Figure 16 and Figure 17 show the experimental results of the method proposed in this paper under the conditions of 3 N·m load and different voltage unbalance.

**Figure 18:**
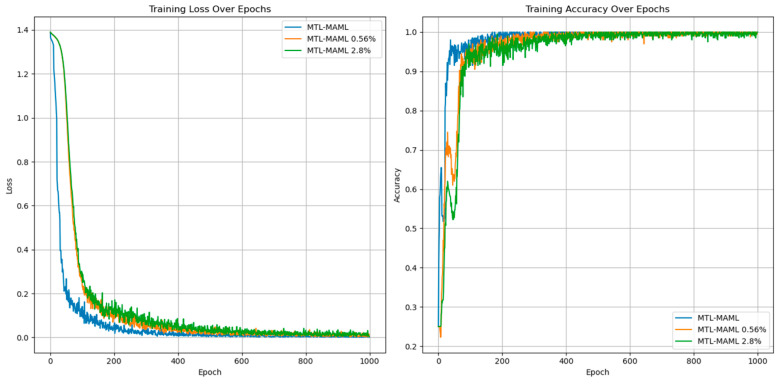
Diagnostic loss value and accurate value under different voltage balance degrees.

As shown in Figure 18, the information indicates that compared with fault diagnosis under different voltage balance states, with the increase in voltage unbalance, the loss value in the corresponding state will increase, and the diagnostic accuracy will decrease. The fluctuation range will also become more intense. The loss value correspondingly increases, and the accuracy rate is stable at 99.45 ± 0.21%, as shown in Table 9.

**Figure 19:**
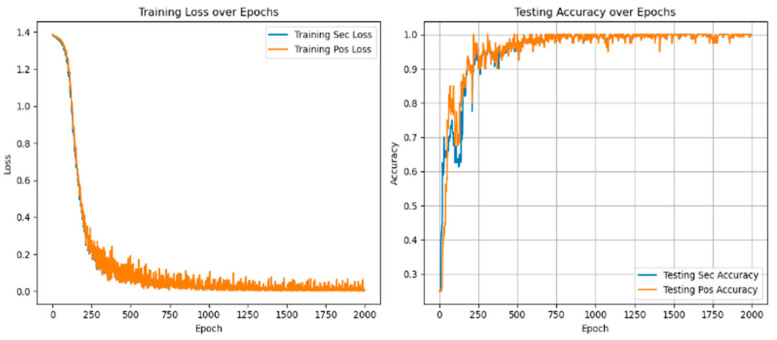
Diagnostic loss and accuracy under voltage balance.

**Figure 20:**
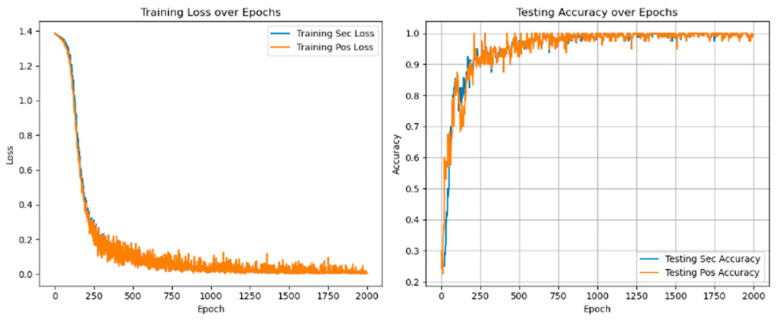
Diagnostic loss and accuracy at 0.56% voltage unbalance.

**Figure 21:**
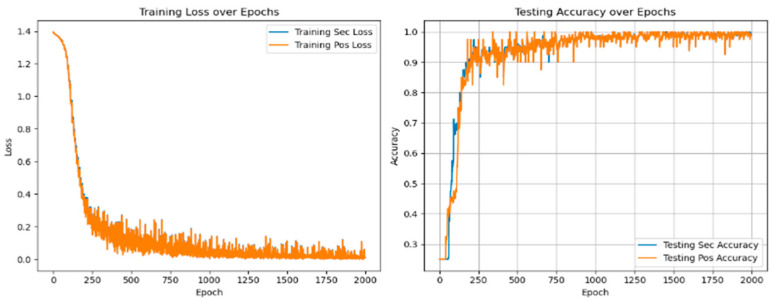
Diagnostic loss and accuracy at 2.8% voltage unbalance.

As shown in Figure 19, Figure 20 and Figure 21, it will be the experimental loss value and accuracy result of PMSM’s fault diagnosis for fault degree and fault location simultaneously under the conditions of a load of 5 N*m and voltage unbalance of 0%, 0.56%, and 2.8%.

**Figure 22:**
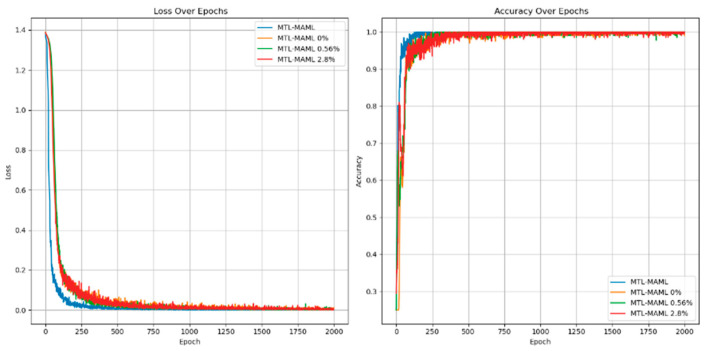
Loss value and accuracy of diagnosis under different voltage balance degrees.

Experiments with different voltage unbalance degrees were carried out under a 5 N*m load. The information in the Figure 22 clearly shows that compared with fault diagnosis under different voltage balance states, as the voltage unbalance degree increases, the loss value under the corresponding state will increase, and the diagnostic accuracy will decrease. The fluctuation range will also become more intense. The loss value correspondingly increases. The accuracy rate is stable at 99.75 ± 0.25%, as shown in Table 10 below.

Under 3 N*m and 5 N*m load conditions, relevant experimental results of different voltage unbalance degrees were carried out, as shown in Figure 22. As can be seen from the figure, the voltage unbalance degree and load changes will affect the loss value and diagnostic accuracy in a small range. Each time the experimental conditions of voltage unbalance degree were changed, the fluctuation range of diagnostic accuracy was not more than 0.74%. Each time the experimental load conditions were changed, the fluctuation range of diagnostic accuracy was not more than 1.02%. The above experimental content fully shows that this method has a good diagnostic effect on PMSM-ITSF under different loads and different voltage unbalance degrees and also has good generalization performance in new environments.

### 4.4. Knowledge Graph of PMSM-ITSF Fault Diagnosis

**Figure 23:**
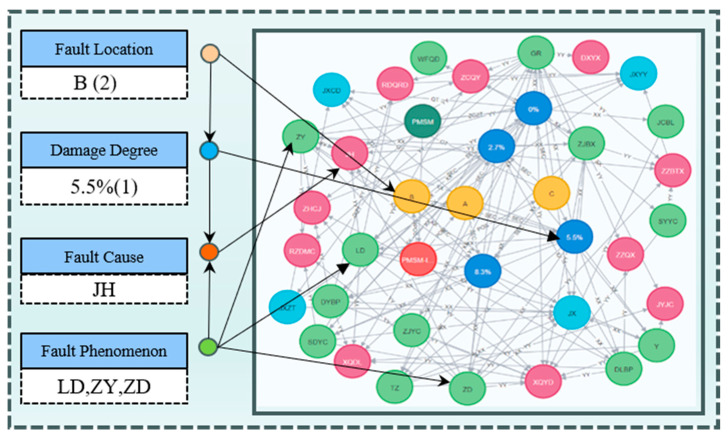
PMSM-ITSF Fault Diagnosis Knowledge Graph.

The above content has comprehensively analyzed the fault characteristic quantity and selected the characteristic quantity of the early inter-turn short circuit fault location and fault degree evaluation. According to PMSM comprehensive fault characteristics, data labels are shown in Table 2. Knowledge graph technology is introduced to conduct the PMSM-ITSF knowledge graph system integrating fault location, degree, symptom, and cause, as shown in Figure 23. Fault diagnosis tasks are completed based on the graph. The spectrum system is composed of 38 nodes and 133 relationships. ITSF location and fault diagnosis of degree are carried out by MTL-MAML, and the fault location, fault degree, fault phenomenon, and fault cause of stator short circuit in PMSM are realized by combining the causal knowledge of PMSM. Among them, the fault phase node is used to locate the fault coil, the fault evaluation node is used to estimate the fault degree of short circuit turns, and the fault phenomenon node and the fault cause node are used to speculate whether the fault cause and effect are caused by the direct cause, indirect cause, or the coupling effect of a direct and indirect cause. This helps the maintenance personnel carry out maintenance quickly and accurately. When MTL-MAML diagnoses the fault signal according to the captured operating data, it can accurately and efficiently lock the “B phase (2)” fault location, quickly assess the “5.5% (1)” fault degree, and deduce the cause of PMSM-ITSF “JH” fault based on the causal knowledge of the fault phenomenon of “LD, ZY, ZD”. In addition, the knowledge graph quickly excludes false signals characterized by the external environment. Based on the analysis results, targeted preventive measures and solutions can be formulated. This will effectively reduce the incidence of PMSM-ITST failure and improve the motor’s operational reliability and service life.

## 5. Conclusions

To solve the problem that the fault sample data of PMSM-ITSF of the industrial robot end effector are small and sparse, and it is difficult to quickly and accurately identify the fault location, evaluate the fault degree, and reason for the fault cause, this paper proposes a multi-task causal knowledge fault diagnosis method of PMSM-ITSF based on ML, which includes two parts. One part is to extract PMSM-ITSF fault text data rules and construct a fault rule chain to build a PMSM-ITSF fault information knowledge base. Another part uses simulation models to simulate datasets of different fault states based on the design of fault indicators with electromagnetic torque, three-phase current, and positive and negative sequence current; multi-task synchronous diagnosis of fault location and fault degree under minor sample conditions is carried out. Experiments show that this method can achieve synchronous diagnosis of PMSM-ITSF fault degree, location, and cause with different voltage unbalanced degrees. The diagnosis accuracy of fault degree can reach 99.75 ± 0.25%, and the accuracy of fault location and fault degree can reach 99.45 ± 0.21%.

The fault diagnosis of industrial robot end effector PMSM-ITSF entirely depends on a vast amount of high-quality datasets. When fault diagnosis is carried out in different cross-modes, the problem of sparse and unbalanced small samples frequently emerges, and it is difficult to obtain a good fault diagnosis accuracy. Limited by the experimental conditions of the physical motor, this paper mainly simulated the experimental dataset by building a mathematical model. Although the simulation can accurately control variables such as the degree of short circuit and load conditions, and repeated experiments are of great value in verifying the effectiveness of the method, it is still necessary to admit that there may be some differences between it and the real experimental data. Therefore, future studies will use physical entity experimental data for training, validation, and optimization of simulation models to improve their consistency with real-world scenarios. In addition, it is also necessary to obtain enough sparse and balanced datasets from the aspects of real experimental data enhancement, data expansion, and combining knowledge expert databases so as to realize online fault diagnosis in different cross-modes of PMSM-ITSF, which will have important research value and potential.

## Figures and Tables

**Table 1:** PMSM Parameters.

Argument	Value
Rated power/W	2200
Rated voltage/V	380
Rated speed/(r/min)	1500
Number of poles	4
Number of turns in parallel winding	252
Stator slots	36

**Table 2:** PMSM Running status value.

	Fault Location	Phase A (1)	Phase B (2)	Phase C (3)
Motor State				
Health (0%) (0)		(0, 0)	(0, 0)	(0, 0)
Slight (2.7%) (1)		(1, 1)	(1, 2)	(1, 3)
Critical (5.5%) (2)		(2, 1)	(2, 2)	(2, 3)
Damaged (8.3%) (3)		(3, 2)	(3, 2)	(3, 3)

**Table 3:** Fault characteristic quantity data distribution.

$i_{A}$	$i_{B}$	$i_{C}$	$i_{g}$	$T_{e}$	Deg	Loc
0.0061	0.9298	0.8567	0.1028	0.5831	0	0
0.2242	0.8858	0.6405	0.1283	0.7592	0	0
0.2681	0.6369	0.7383	0.3579	0.6419	1	1
0.5373	0.5077	0.6301	0.6089	0.3198	1	2
0.9686	0.2900	0.1603	0.8200	0.2951	1	3
0.7391	0.6711	0.5836	0.5781	0.4733	2	1
0.6949	0.7452	0.4146	0.8704	0.4719	2	2
0.6251	0.4799	0.4194	0.7577	0.4808	2	3
0.8314	0.1844	0.3392	0.7800	0.1436	3	1
0.7945	0.2756	0.1891	0.8639	0.1394	3	2
1.0000	0.0000	0.0015	1.0000	0.0446	3	3

**Table 4:** PMSM-ITST Different algorithms for fault degree support set diagnostic accuracy.

Sample	Method (Sec)	4-Way Accuracy Rate		
		1-Shot	2-Shot	5-Shot
1200	MTL-DL	68.02 ± 0.89%	71.49 ± 0.67%	72.65 ± 0.53%
1200	MTL-MetaSGD	87.95 ± 0.83%	90.75 ± 0.62%	94.32 ± 0.49%
1200	MTL-MAML	94.65 ± 0.77%	97.47 ± 0.59%	**98.84 ± 0.43%**

**Table 5:** PMSM-ITST Different algorithms of fault degree query set diagnosis accuracy.

Sample	Method (Sec)	4-Way Accuracy Rate		
		5-Shot, 1-Query	5-Shot, 2-Query	5-Shot, 5-Query
1200	MTL-DL	72.02 ± 0.82%	72.53 ± 0.59%	73.5 ± 0.44%
1200	MTL-MetaSGD	90.07 ± 0.79%	93.25 ± 0.53%	97.08 ± 0.38%
1200	MTL-MAML	97.3 ± 0.65%	98.61 ± 0.44%	**99.75 ± 0.25%**

**Table 6:** PMSM-ITST Different algorithm diagnosis accuracy of fault degree.

Sample	Method (Sec)	Accuracy Rate
1200	MTL-DL	73.5 ± 0.44%
1200	MTL-MetaSGD	97.08 ± 0.38%
1200	MTL-MAML	**99.75 ± 0.25%**
1200	GAN [54]	90.00%
6000	Cycle GAN-DAE [33]	98.84%
16,500	GAN-SAE [54]	99.40%
16,384	CGAN-CNN [55]	99.50%

**Table 7:** Comparison of loss value and accuracy of single-task learning and multi-task learning.

Learning Type	Fault Type	Training Accuracy	Verification Accuracy
Single-task learning	Degree of failure	97.45 ± 0.49%	98.53 ± 0.35%
	Fault location	92.53 ± 0.95%	94.39 ± 0.83%
Multi-task learning	Degree of failure	98.36 ± 0.37%	**99.75 ± 0.25%**
	Fault location	97.41 ± 0.78%	98.75 ± 0.59%

**Table 8:** Fault degree and fault location of PMSM-ITST diagnosis accuracy of each algorithm.

Sample	Method (Sec)	4-way ACCURACY		
		5-Shot, 1-Query	5-Shot, 2-Query	5-Shot, 5-Query
1200	MTL-DL	70.85 ± 0.66%	71.53 ± 0.54%	72.07 ± 0.29%
1200	MTL-MetaSGD	90.16 ± 0.53%	92.95 ± 0.51%	95.54 ± 0.34%
1200	MTL-MAML	97.43 ± 0.51%	98.11 ± 0.42%	**99.45 ± 0.21%**

**Table 9:** The diagnostic accuracy of different voltage unbalance degrees with 3 N*m load.

Sample	Method	Different Voltage Unbalance Degree		
		0%	0.56%	2.8%
1200	MTL-MAML	99.45 ± 0.21%	99.24% ± 0.47%	98.5 ± 0.59%

**Table 10:** The diagnostic accuracy of different voltage unbalance degrees with 5 N*m load.

Sample	Method	Different Voltage Unbalance Degree		
		0%	0.56%	2.8%
1200	MTL-MAML	99.25 ± 0.26%	98.54 ± 0.44%	97.83 ± 0.57%

## Data Availability

Data are contained within the article.

## Abbreviations

The following abbreviations are used in this manuscript:

PMSM	Permanent Magnet Synchronous Machine
ITSF	Inter-Turn Short-Fault
ML	Meta-Learning
DL	Deep Learning
Cycle GAN	Cycle Generative Admissible Network
DAE	Deep Autoencoder
MTL	Multi-Task Learning
PSC	Positive-Sequence Current
NSC	Negative-Sequence Current
KG	Knowledge Graph
